# *Cyanea capillata* Bell Kinematics Analysis through Corrected *In Situ* Imaging and Modeling Using Strategic Discretization Techniques

**DOI:** 10.1371/journal.pone.0115220

**Published:** 2014-12-26

**Authors:** Alex A. Villanueva, Shashank Priya

**Affiliations:** Bio-inspired Materials and Devices Lab (BMDL), Center for Energy Harvesting Materials and Systems (CEHMS), Virginia Tech, Blacksburg, Virginia, United States of America; Scientific Institute Foundation Santa Lucia, Italy

## Abstract

Obtaining accurate kinematic data of animals is essential for many biological studies and bio-inspired engineering. Many animals, however, are either too large or too delicate to transport to controlled environments where accurate kinematic data can be easily obtained. Often, *in situ* recordings are the only means available but are often subject to multi-axis motion and relative magnification changes with time leading to large discrepancies in the animal kinematics. Techniques to compensate for these artifacts were applied to a large jellyfish, *Cyanea capillata,* freely swimming in ocean waters. The bell kinematics were captured by digitizing exumbrella profiles for two full swimming cycles. Magnification was accounted for by tracking a reference point on the ocean floor and by observing the *C. capillata* exumbrella arclength in order to have a constant scale through the swimming cycles. A linear fit of the top bell section was used to find the body angle with respect to the camera coordinate system. Bell margin trajectories over two swimming cycles confirmed the accuracy of the correction techniques. The corrected profiles were filtered and interpolated to provide a set of time-dependent points along the bell. Discrete models of the exumbrella were used to analyze the bell kinematics. Exumbrella discretization was conducted using three different methods. Fourier series were fitted to the discretized models and subsequently used to analyze the bell kinematics of the *C. capillata*. The analysis showed that the bell did not deform uniformly over time with different segments lagging behind each other. Looping of the bell trajectory between contraction and relaxation was also present through most of the exumbrella. The bell margin had the largest looping with an outer path during contraction and inner path during relaxation. The subumbrella volume was approximated based on the exumbrella kinematics and was found to increase during contraction.

## Introduction

Jellyfish have a unique propulsion mechanism that is of interest to a diverse group of researchers from disciplines such as biology [1], [2], engineering [3], [4], and oceanography [5]. The mechanism by which jellyfish generate thrust in combination with other essential functions required to survive can provide insight for a variety of applications such as vehicle propulsion, energy harvesting, synthetic heart valves and animal foraging. Our interest lies in using jellyfish as inspiration for the development of unmanned underwater vehicles (UUVs) [6], [7]. This would provide low cost of transport vehicle with silent operation. The realization of this vision is dependent upon the fundamental understanding of the mechanics, hydrodynamics and fluid-structure interaction around large dimension jellyfish.

**Figure 1:**
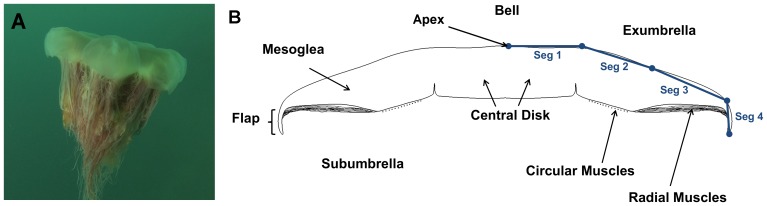
*Cyanea Capillata.* (A), image of a *Cyanea capillata* in the relaxed position. (B), Schematic of a *C. capillata* bell cross-section showing different anatomical parts and a four-segment model of the exumbrella.

**Figure 2:**
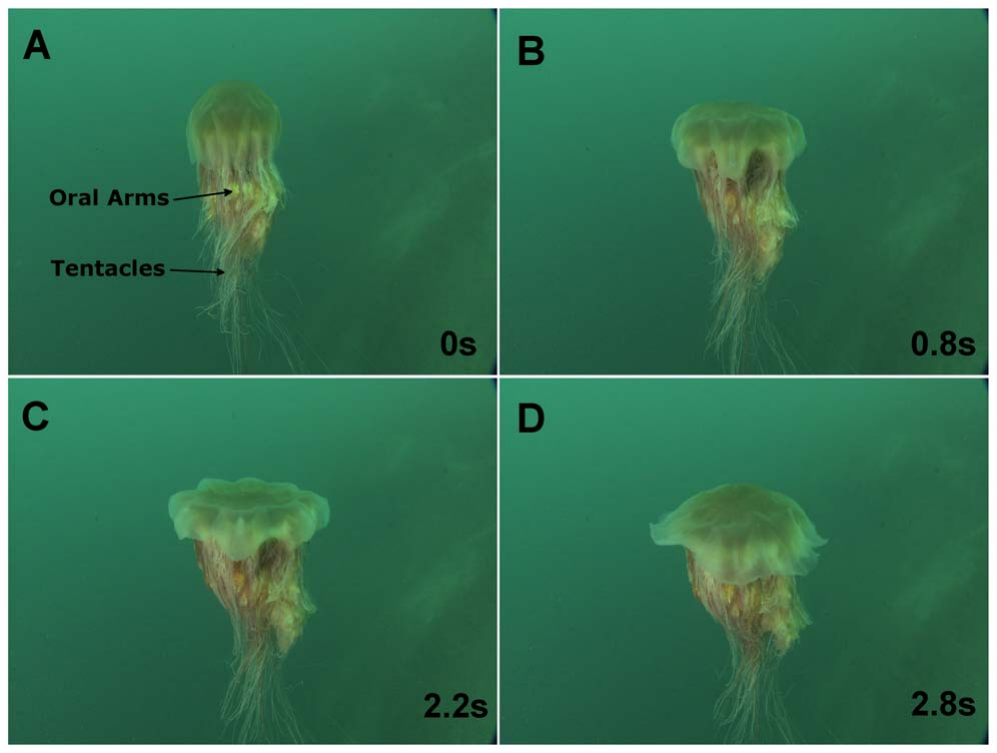
*Cyanea Capillatas* wimming sequence. Swimming sequence of a large *C. capillata* in the Atlantic Ocean off the Norwegian shore. The animal is shown in the (A) fully contracted, (B) relaxing, (C) fully relaxed and (D) contracting states. The swimming cycle shown is the first of two processed cycles.

*Cyanea capillata*, see Fig. 1A, are found in the North Atlantic, Pacific and Artic Oceans [8]. *C. capillata* are usually of reddish-brown or yellowish color [9]. They can reach dimensions exceeding 2 m in bell diameter and tentacles of 36 m long [10]. *C. capillata* have one significant difference from most other jellyfish species; they have a segmented bell as opposed to a uniform bell. Segmentation allows flaps surrounding each of the bell segments to fold on itself during relaxation and expand during contraction. A maximum form area is therefore achieved during contraction and minimized during relaxation. Circular muscles in jellyfish subumbrella are typically solely responsible for propulsion. The *C. capillata* uses a combination of circular and radial muscles to contract its bell and create propulsion [11]. Gladfelter reported that the circular muscles contract the bell for the first 30°–45° below the horizontal and the radial muscles further contract the bell up to a total of 90°. The *C. capillata* tentacles originate from the subumbrella and are used to capture prey as they come in contact with the nematocysts on the tentacles, see Fig. 2A. The subumbrella also has four oral arms, see Fig. 2A, with length of about a bell diameter [8], [10].

It is important to understand the implications of size on the propulsion mechanism of jellyfish. However, there is very little information available in literature addressing this issue. McHenry and Jed [12] investigated the scaling effects of *Aurelia aurita* on hydrodynamic performance. Their specimen diameters ranged from 1.57 to 9.51 cm. Herschlag and Miller [13] have studied the effect of Reynolds number on *A. aurita* specimen ranging from 0.36 to 10.2 cm in diameter. *C. capillata* can reach dimensions over an order of magnitude from what has been analyzed and there is currently no hydrodynamic analysis of such large jellyfish.

Jellyfish swim in an oscillating fashion which consists of a two phase cycle: contraction and relaxation. Contraction is achieved by muscles located in the subumbrella as shown in Fig. 1B, which causes a circumference decrease. The bell thickness then increases non-linearly and strains the radial mesogleal fibres [14]. These fibres store elastic energy which is used to passively relax the bell to its original geometry. Jellyfish can be separated into two main categories based on their propulsion mechanism: rowing and jetting. Rowing jellyfish are oblate and can reach large dimensions, while jetting jellyfish are prolate and smaller [15]. Rowers and jetters produce a starting vortex during contraction. During relaxation, both refill their bells creating a stopping vortex in the subumbrella. Rowers utilize the interaction between the starting and stopping vortices to create more thrust and have a larger efficiency which enables them to reach larger dimensions [16]–[18]. Several investigations have been conducted regarding the bell kinematics of rowers [1], [15], [19]–[23], however, none have provided a time dependent model which can be used to replicate the jellyfish kinematics of the whole bell over a full cycle. This is often required to model the structural mechanics, hydrodynamics and mechanism. In this work, a discrete kinematic exumbrella model is developed and is used to analyze the function of the different anatomical features responsible for propulsion.

Jellyfish bell kinematics vary significantly from other animals. The locomotion is performed by a bell that is nearly uniform in structure, has high flexibility and undergoes large curvature change. This is different than most animals where motion occurs by actuating rigid internal structures about a pivot point such as in a bone and joint mechanisms. The lack of rigid structures demands new kinematics tracking and analysis methods. Flexible structures can be discretized in order to estimate their deformation and motion. Jellyfish bells have previously been discretized for computational fluid dynamics (CFD) applications [18], [13], [24]. In those instances, nodes are prescribed a certain displacement or force and the structure's resulting kinematics interacts with surrounding fluid.

The ability to analyze in situ video from field recordings is crucial for the study of many organisms which are either too large or too delicate to work within the laboratory environment. In addition, field recordings allow observation of animals in their natural habitat, thus reducing the probability of behavioral alterations and other effects common in laboratory studies such as wall effects [25]. High quality kinematic data is required for many biological studies as well as for the development of biomimetic and bio-inspired systems. In a controlled environment, two-dimensional (2D) analysis can often provide suitable kinematics of a specimen [20], [26]–[31]. Three-dimensional (3D) methods, such as multiple integrated 2D cameras can capture more complex kinematics where substantial motion is occurring in all three planes [32]–[34]. However, these techniques are difficult to apply in most field settings where animal locations are often random and their presence is spontaneous. In these situations a single camera operated by hand often provides the only means for recording the animal behavior. This problem is exemplified in situations where a free-swimming scuba diver needs to record large free-swimming animals such as fish, marine mammals, sea turtles or jellyfish. In this scenario, not only is the animal moving freely in 3D but so is the diver.

In this study, we present a novel method for obtaining accurate kinematics from in situ underwater recordings. We apply the method to a recording of a large *C. capillata* to analyze its kinematics. Large *C. capillata* cannot feasibly be analyzed in a controlled environment and as a result, little information is available in the literature on their behavior and swimming kinematics. These techniques will help in deriving accurate kinematics from video recordings obtained in sub-optimal conditions. This provides the ability to extract potentially valuable biological information from existing footage and future recordings.

## Materials and Methods

### Correction of Bell Kinematics

An in situ video of a large *C. capillata* was used to elucidate the swimming kinematics of the animal. The video was obtained from the Ocean Footage video library [35] and was filmed in the Atlantic Ocean off the coast of Norway. A swimming sequence of the video is shown in Fig. 2. The video used lasts 30 s and features multiple swimming cycles. The exact animal size in the video is unknown but is approximated to be 50 cm in diameter using a diver nearby as reference.

The kinematic analysis can be visualized to take place on a 2D surface perpendicular to the camera. Traditionally, in order to capture the animal kinematics accurately, several criteria should be met: (1) The animal motion must occur in a 2D plane parallel to the camera plane. For animals that are not axi-symmetric such as the *C. capillata*, the angle that the animal faces the camera is important. The *C. capillata* consists of eight radial bell segments and two opposing bell segments should be perpendicular to the field of view to extract the desired bell kinematics. (2) The 2D plane must be at a constant distance from the camera during the duration of the analysis. Otherwise, the animal scale changes over time. (3) The animal must not rotate about the x- or y-axis of the camera. Rotation out of plane causes part of the profile information to be lost when projected onto the 2D camera plane. The later criterion cannot be compensated during post-processing if only one view of the motion is available.

The first step in capturing the *Cyanea capillata* bell kinematics is (i) profile tracking of the bell. This can be achieved by tracking the exumbrella profiles since they provide most of the bell kinematics and can be detected from the high contrast formed with the background. The captured exumbrella profiles need to be corrected in order to provide useful kinematic information. The second step is to (ii) body tracking and removing the overall body motion from the bell kinematics. The third step is to (iii) remove any magnification occurring during the video sequence to prevent a change of scale in the kinematics. The fourth step is to (iv) remove body rotation and the fifth step is (v) strategic bell discretization for kinematic analysis.

#### Profile Tracking

**Figure 3:**
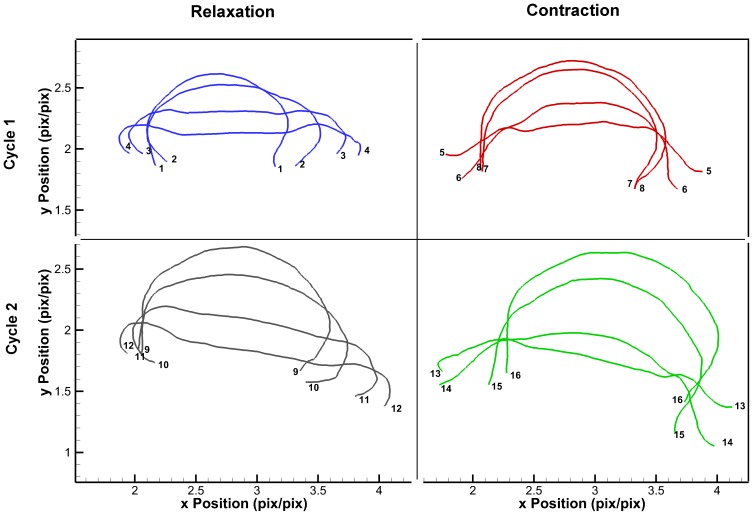
Exumbrella profiles during swimming. *Cyanea capillata* exumbrella profiles over two full swimming cycles before processing. Profile tips are numerically labeled in order of occurrence. Note the change in apparent animal size over just two swimming cycles. The profiles displayed were manually selected in order to fully display the bell kinematics at different instances. Each contraction and relaxation phases for both cycles are represented by a different color. The position has been normalized by the half exumbrella arclength in the relaxed position of the first cycle.

In a dynamic environment such as the ocean, it is difficult to achieve all the aforementioned criteria. The selected video of *C. capillata* met criteria (1) and (3) with negligible rotation about the x- and y-axis but did not meet the criterion (2). The footage begins with the jellyfish's bell fully contracted and covers two full cycles. It was filmed at 25 fps with image resolution of 640×360 pixels. Edge detection was performed manually for the entire exumbrella from one margin to another at every 5 frames using ImageJ resulting in profile points 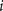 for each frame 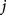. Selected processed profiles are shown in Fig. 3.

#### Body Tracking

The profile points were post-processed in order to obtain a set of half profiles which can be used for kinematic analysis. In order to do so, the bell apex must be found. The apex is the top point of the exumbrella when looking at the bell from its side and the location where the axial axis meets the exumbrella. To find the apex, two different methods were considered since post-processing for analysis strongly relies on the apex location. The first method consists of measuring the exumbrella arclength with the jellyfish in the fully contracted position and then taking the midpoint of the full arclength as the apex. This method leads to a significant error in the apex position due to the complexity in locating the bell margin in the images. Margin location largely affects the exumbrella arclength and therefore, the apex location. The second method considered and used, consists of tracking a visible point on the bell near the apex and then shifting the point by the distance between it and the apex as measured using the first method for the first frame in the cycle. The profile points were divided into two half-profiles originating at the apex and ending at the margin. The half-profiles were than normalized by the exumbrella arclength of the half-profile in the relaxed position of the first cycle.

#### Magnification

The chosen image sequence was recorded from a camera that moves relative to the animal. The animal itself was moving relative to the ocean floor. These factors affected the scale of the animal throughout the video and had to be accounted for in order to resolve the bell kinematics. The easiest approach would be to use an animal feature that has a fixed dimension throughout the video and scale relative to it. However, there are no such features on the jellyfish that can be used for this approach. Therefore, two other techniques were used to account for magnification.

**Figure 4:**
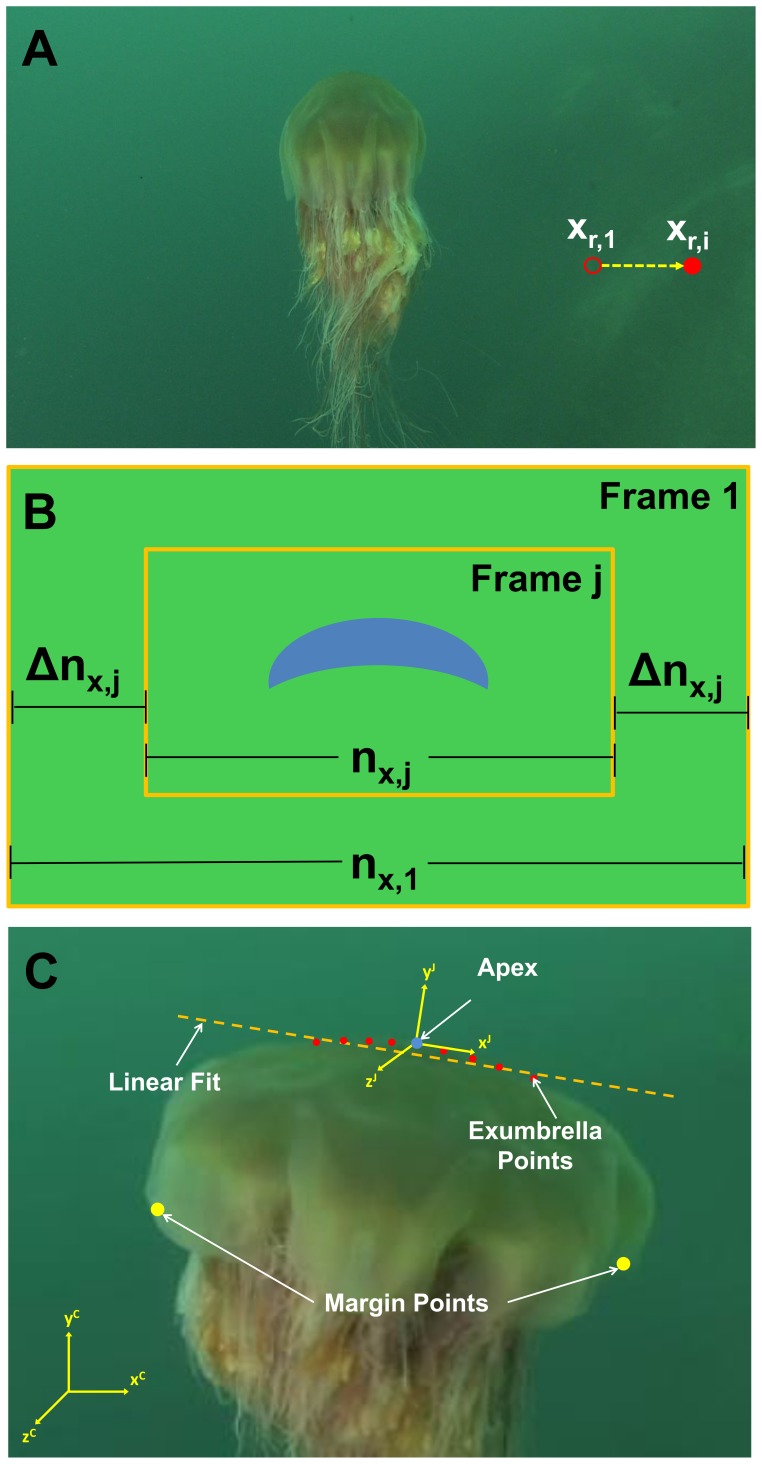
Correction technique illustration. Illustrations of how image magnification was compensated by using a reference point on the ocean floor. (A) Frame of the *C. capillata* footage with reference rock highlighted by a red hollow dot in the first frame and red dot in frames j>1. (B) Schematic of magnification correction technique using a background reference point. (C) Schematic of the method used to account for the body rotation about the 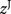-axis and 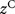-axis. The red dots represent the exumbrella points 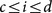 which meet the 24% criteria. The linear fit was calculated from these exumbrella points.

The first method (*a*) uses the reference in the background to estimate the motion of the camera relative to the background. Significant image magnification occurs in the image sequence which is due to the camera moving relative to the ocean floor. A rock on the ocean floor was tracked and used to estimate the width variation in the image plane resulting from magnification. This is depicted in Fig. 4A where the rock moves out of frame as the camera moves forward.

The total number of pixels is constant for each frame. Therefore, the following is true in the x-direction:
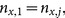
(1)where 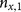 and 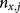 are the number of pixels in frame 1 and j respectively. Frame 1 is taken as the reference frame from which the rest of the frames will be adjusted to match its scale. Eq. 1 is illustrated in Fig. 4B. As the jellyfish is magnified in the camera frame, the rock moves out of frame by a distance of:
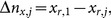
(2)where 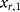 and 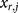 are the x-position of the reference rock in frame 1 and 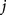 respectively. The magnification results in an increase in resolution where more pixels represent the jellyfish. This increase in pixel is proportional to the rock displacement by:
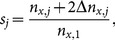
(3)where 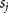 is the scale for frame 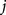 by which the profile coordinates are multiplied in order to keep the same scale as in the reference frame. Eq. 3 assumes that the camera moves with the jellyfish centered in its frame. The displacement of the rock in the y-direction is negligible in this image sequence. The same analysis could be conducted with displacement in the y-direction. The analysis also assumes that the camera is moving only in the z-axis. Displacement in the x-axis or rotation about the y-axis of the camera would result in error.

The second method (*b*) uses a varying dimension on the animal at the same position in different swimming cycles. The *C. capillata* specimen used in this study and other common jellyfish don't have anatomical features of fix length that can be used as a reliable reference point for determining the magnification. The total exumbrella arclength can be used as a size reference but the exumbrella naturally increases in length during contraction and decreases in length during relaxation. This is due to the swimming muscles compressing the subumbrella and thereby causing a slight expansion of the exumbrella [36], [37]. Therefore, the arclength cannot be used directly to scale the images. Instead, the exumbrella arclength is compared at a given stage of different swimming cycles. Costello and Colin [1], [19] have studied the basic bell kinematics of the *Cyanea capillata* and other jellyfish species over several swimming cycles. They have shown that the jellyfish swimming kinematics are cyclical and repeatable during straight swimming. We can therefore assume that the bell will have the same length at a given point during a cycle. The most accurate points and easiest to locate in a given cycle are found during a full contraction or full relaxation. This usually corresponds to a minimum and maximum diameter respectively or the geometry right before the relaxation or contraction respectively. The exumbrella arclength in the fully contracted position was used at three different times in the swimming sequence. The exumbrella arclength is calculated using: 
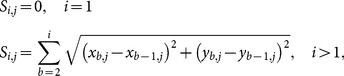
(4)where 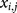 and 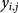 are the coordinates of the exumbrella point 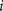 on frame 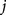. To account for the displacement of the jellyfish relative to the camera, the animal itself is used as a reference.

#### Body Rotation

Body rotation is quantified for analysis and for correction. Two coordinate systems are established to account for body rotation. The first coordinate system labeled C, is fixed on the camera lenses and the second coordinate system labeled J, is a body fixed coordinate system which is centered on the jellyfish apex as shown in Fig. 4C. The *C. capillata* specimen rotates about the 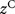-axis during its two swimming cycles. We assume that the 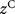-axis and 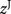-axis are parallel. In this application, the rotation angle is calculated so it can be removed from the bell kinematics. In other words, we want to analyze the jellyfish motion from the jellyfish coordinate system.

Two different methods were considered to calculate body rotation of the jellyfish relative to the camera coordinate system. The first method uses the angle formed by a line passing through the margin points. As the animal stabilizes itself, it undergoes small non-axisymmetric kinematics. This causes the margin to deform at different rates on each side and therefore causes this method to result in incorrect angles. Also, the slightest discrepancy in margin position will significantly affect the angle. The second method uses the fact that the area in proximity of the apex undergoes very small deformation during swimming and is also not affected significantly by non-axisymmetric deformation [15], [22], [23]. A linear fit of points in that region is used to produce a line from which the angle of the animal is calculated as shown in Fig. 4C. The points falling within ±24% of the exumbrella arclength on each side where 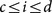, were selected for this calculation. 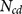 is the total number of points from 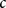 to 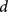. The mean location of these points was calculated as follows:
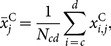
(5)

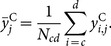
(6)


A linear fit was calculated using the following equation:
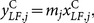
(7)where:
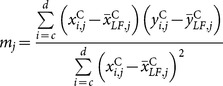
(8)and 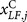 and 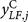 are the components of the linear fit. The rotation angle about the 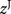-axis is:
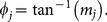
(9)


The exumbrella points in the jellyfish coordinate system are:
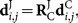
(10)where:
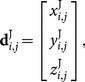
(11)

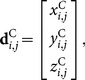
(12)are the position vectors for each point in the jellyfish and camera coordinate systems respectively and:
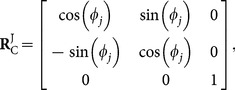
(13)is the rotation matrix to convert between the camera and jellyfish coordinate system. The profiles were then divided into two half-profiles, left and right, with apex as the root of each hald-profile. The profile points were then filtered with a second order Butterworth low pass filter with cut-off frequency of 20% of the Nyquist frequency which varied from 0.28 to 0.44 samples per pixel. This filter was applied to the profile points after converting to polar coordinates which was necessary due to the curvature of the bell. The points were then converted back to Cartesian coordinates for analysis.

#### Profile Discretization by Interpolation

A random number of points were collected for each profile during edge detection. A fixed number of points for each profile are desired to allow the analysis of a given point on the exumbrella. Since the exumbrella deforms during a swimming cycle, this method assumes it deforms equally throughout its arclength. A cubic spline was fitted to each half profile and by interpolating points b for a total of N_b_ = 51 points per profile. The points span from the apex b = 0 to the margin b = 51. A description of cubic spline interpolation can be found in [38].

### Strategic Bell Discretization

A model of the *C. capillata* exumbrella can be achieved by discretizing the bell by finding strategic node locations that meet two criteria: (i) highest deformation as a function of arclength and (ii) highest deformation as a function of time. Three discretization methods were explored to locate nodes which meet the two criteria. (1) The curvature method uses a series of exumbrella profile to determine where most of the deformation occurs over time. (2) The anatomical method consists of observing the animal's anatomy to identify where the variations in structure and anatomical features might affect deformation. (3) The node optimization method consists of optimizing the node location for a set of segments by minimizing the error found between the discrete model and natural profiles.

#### Curvature

Curvature is calculated using the definition of a circumcircle which is: a circle passing through three vertices F, G and H of a triangle with sides of length f, g and h. The diameter of a circumcircle is:
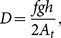
(14)where *A_t_* is the area of the triangle. The area can be found using Heron's formula: 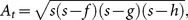
(15)where:
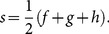
(16)


The curvature of the circumcircle is:
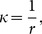
(17)where 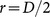 is the circumcircle radius. The curvature equation for a discrete curve in Cartesian coordinates becomes:
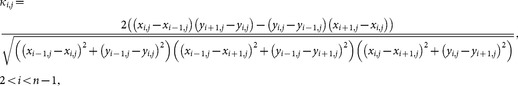
(18)where *n* is the total number of points for a given exumbrella profile. The derivative of curvature with respect to arclength was taken as follows: 
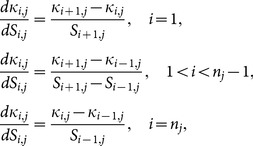
(19)where the arclength at each point 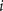 is calculated using equation (4). Potential node locations can be found by setting the curvature derivative in Eq. 19 equal to zero. This identifies the exumbrella locations where curvature varies most as a function of arclength and meets criteria (i). The second criterion is met by looking at the standard deviation of potential node locations as a function of time. The variance is calculated as:
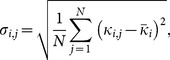
(20)where 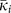 is the mean curvature for a given point.

#### Anatomical Analysis

The second method used to determine node locations consisted of analyzing the anatomical features of the natural animal to determine the musculature and joint arrangements. Gladfelter [11] studied the anatomy of *C. capillata* ranging from 2 to 30 cm in diameter. A specimen laying on its exumbrella with tentacles removed was used to obtain the dimensions of different features. The features of interests were the central disk, circular muscles, radial muscles and flap, see Fig. 1B. The dimensions were taken from the center of the bell to the different features and normalized by the bell radius.

#### Error Analysis

**Figure 5:**
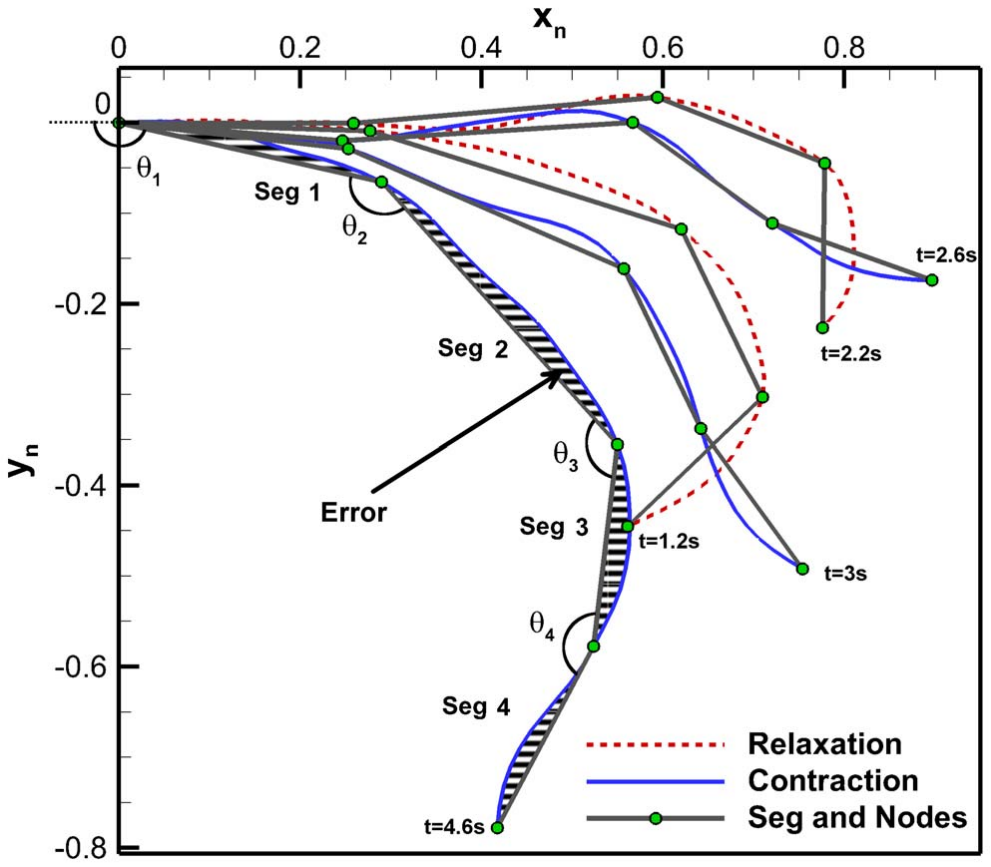
Four-segment model and exumbrella profiles. The four-segment optimized model is overlaid on selected *C. capillata* exumbrella profiles. The time at which each profile occurs from the beginning of the relaxation phase are labeled. The segments and angles formed between each segment are also labeled. The striped area shows the error between one exumbrella profile and corresponding model.

An error analysis was conducted to determine a segment configuration which best matched the animal's bell kinematics. It also allowed the evaluation of the other two different node detection methods. The area formed between the *C. capillata* exumbrella profile and the exumbrella model, see Fig. 5, was used to quantify the discrepancy between both profiles. This discrepancy or error is calculated using the following:
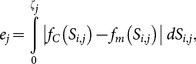
(21)where 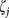 is the total arclength for each exumbrella profile 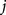, 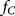 and 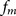 are the functions of exumbrella profiles and discrete exumbrella models respectively. The exumbrella models consist of nodes starting at the apex and ending at the bell margin that are interconnected by lines. The position 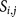 of a profile node and corresponding model node are used to calculate the area formed between both. The error during two cycles was then summed as follows:
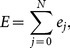
(22)


This gives the total error 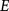 for a given discrete exumbrella model. It should be noted that the error is dimensionless since exumbrella points and arclength are dimensionless.

### Subumbrella Volume Change

Jellyfish propulsion is often modeled as a jetting mechanism where thrust is a function of subumbrella volume change [39]. The subumbrella volume can be approximated using the exumbrella profiles. This approximation neglects the bell thickness between the exumbrella and subumbrella and the tentacles attached to the subumbrella. The mesoglea volume is near constant during actuation and the tentacles are fixed to the subumbrella. Therefore, this method provides a good estimation of the water volume being ejected during contraction. The subumbrella volume is defined as the space delimited by the exumbrella profile and a horizontal line passing through the minimum exumbrella y-position for each profile, 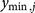. The subumbrella volume for a given profile was calculated using the following equation:
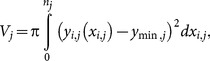
(23)


Equation 23 assumes the *C. capillata* bell is axi-symmetric which is a crude representation of the bell geometry but gives a good approximation for this analysis.

## Results

### Corrected Bell Kinematics

#### Magnification

**Figure 6:**
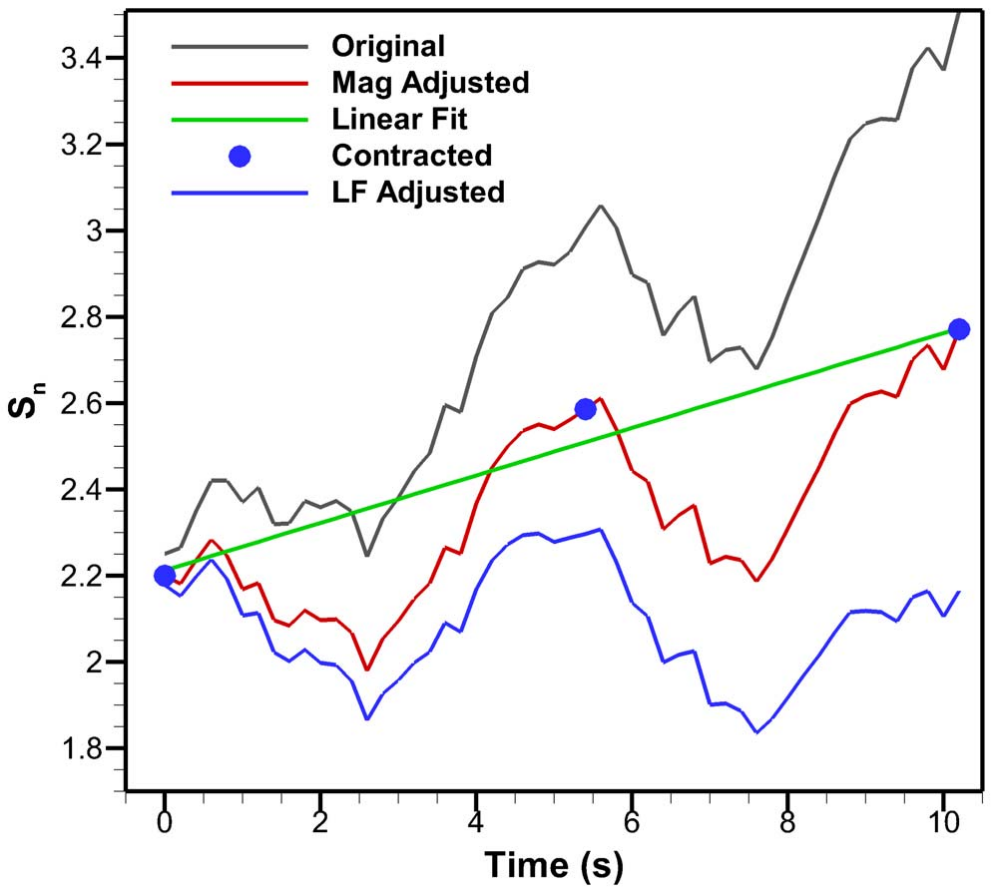
Exumbrella arclength. Normalized exumbrella arclength as a function of time for two full swimming cycles starting fully contracted. The “Original” lengths correspond to the unprocessed profiles. The “Mag Adjusted” lengths are the “Original” lengths corrected for magnification based on the camera movement relative to the ocean floor. The “Linear Fit” is through the three fully “Contracted” instances. The “LF Adjusted” lengths are the “Mag Adjusted” lengths with the additional “Linear Fit” adjustment.

The total exumbrella arclength 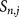 as a function of time is shown in Fig. 6. A sinusoidal pattern is expected as the bell contracts and relaxes. However, the original exumbrella arclength had a net increase after two cycles. Once adjusted for magnification due to the camera moving relative to the ocean floor, the average arclength over time still increased. This was due to the animal's movement relative to the camera and was accounted for by calculating the exumbrella arclength in the fully contracted position at three different times. The contracted positions were taken at the moment before the bell started relaxing and the exumbrella arclength was assumed to be constant at those three instances. A linear fit (LF) was made through those three points and the data was scaled accordingly as shown by the LF Adjusted curve in Fig. 6. This adjustment assumes that the magnification due to jellyfish movement relative to the camera was constant over the two swimming cycles. The adjusted result shows only minor discrepancies that could be due to rotation of the animal during swimming or slight irregularities in the animal's bell motion.

#### Bell Kinematics

**Figure 7:**
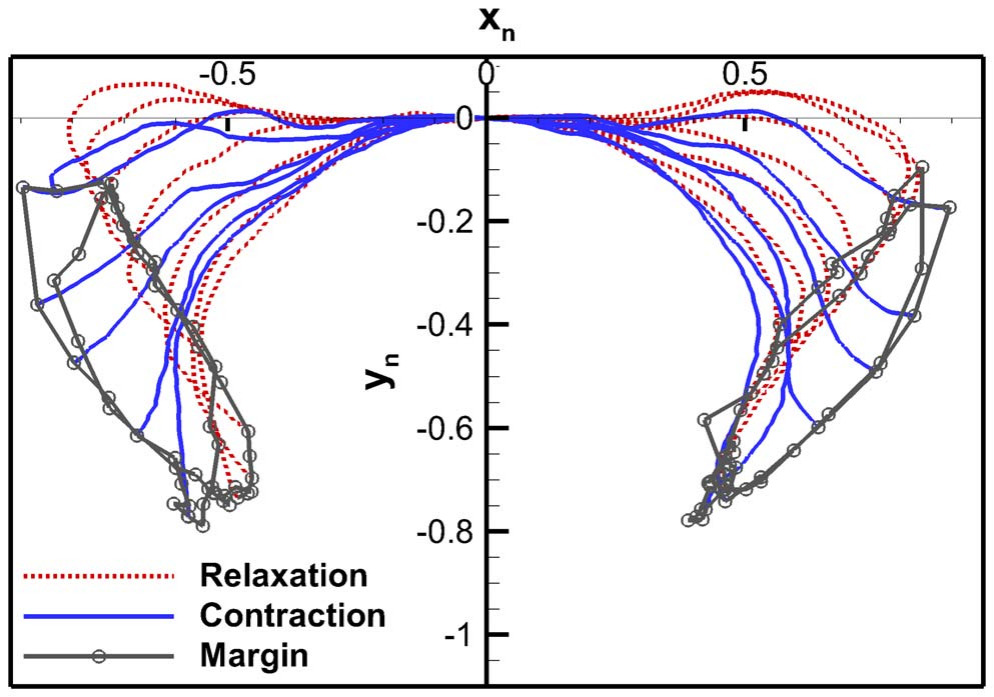
Corrected kinematics. *Cyanea capillata* exumbrella profiles are shown during a full cycle. The profiles were arbitrarily selected over the first cycle of the swimming sequence to demonstrate the different geometries encountered. Bell margin trajectory is shown for two consecutive cycles. The positions are normalized by the half exumbrella arclength in the relaxed position.

Selected exumbrella profiles after processing are shown in Fig. 7 for a full cycle. The bell margin trajectories are also shown for two consecutive cycles. The overlay of the margin trajectories for two cycles show the accuracy at which the correction methods were able to account for different video artifacts in the kinematics.

The margin trajectories for the two cycles overlay well which means the bell is following a similar path during both cycles. In order for this to occur, magnification and rotation had to be properly compensated. Minor variations are seen after processing which can be due to inconsistencies in deformation when the animal is reacting to water currents, minor errors from manual edge detection, the footage not fully meeting criteria (1) and (3) through the two cycles and the assumption of linear motion between the animal and camera.

During relaxation, the bell goes over the apex as seen at x_n_ = 0.5 of Fig. 7. This high relaxation is not common in most jellyfish [1], [15], [19], [20], [22]. Also, most of the motion occurs in the y-direction with an average distance for both cycles and each side of y_n_ = 0.62 and x_n_ = 0.28 at the bell margin between the relaxed and contracted positions. The flap flares outwards during contraction and is bent inwards during relaxation. This results in an outer trajectory during contraction and inner trajectory during relaxation. The same observation has been made with *A. aurita* [23].

### Strategic Bell Discretization

#### Anatomical Method

The anatomical analysis revealed five important locations which set delimitation for a four-segment model. The 0% location corresponds to the bell apex which is at the center of the central disk. The central disk region is passive and consists of mesoglea and gonads. It is delimited by the coronal joint and circular muscles at 40%. This joint eases deformation at that location when the circular muscles actuate. The radial muscles begin at the outer edge of the circular muscles which is at 64%. This location of actuator transition will be less stiff then the surrounding area due to the lack of muscles. It is therefore also considered as a node location. The flap starts where the radial muscles end at 88%. This is a location of mechanical transition from active to passive. The flap is also more flexible due to its lack of muscles which will result in more compliance during contraction and relaxation. The fifth node is taken as the bell margin 100%, which also marks the end of the flap.

#### Curvature Method

**Figure 8:**
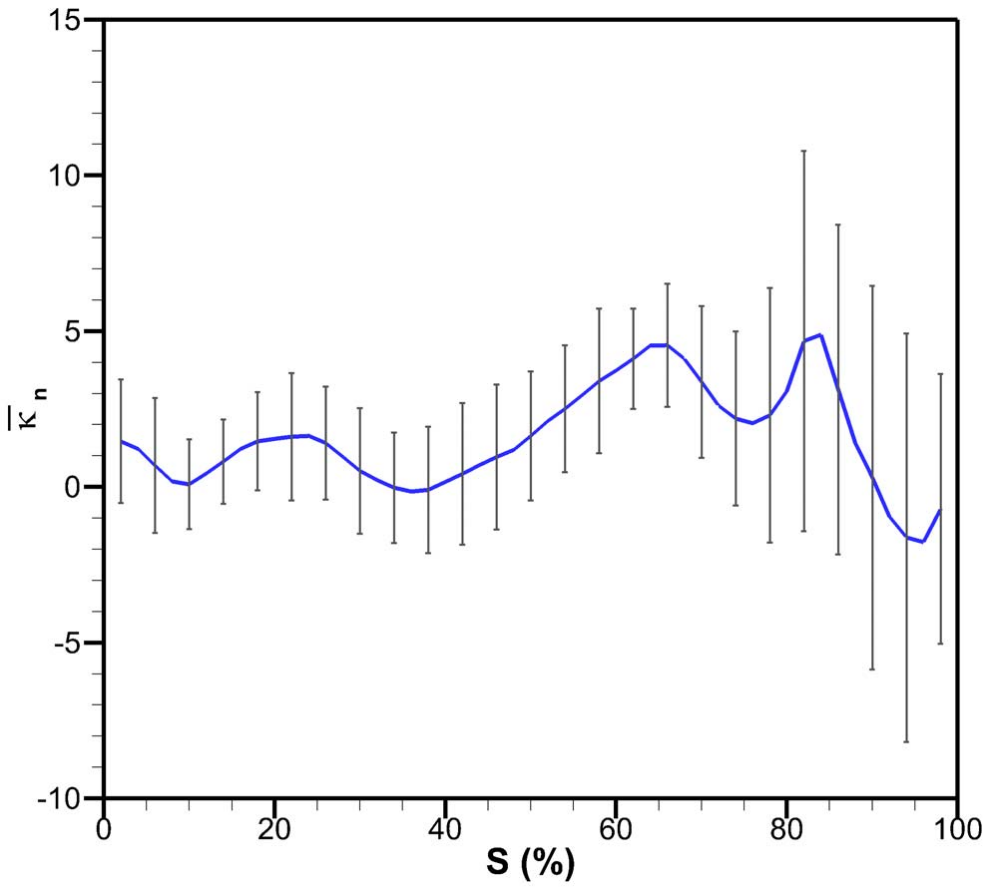
Exumbrella curvature. Average curvature as a function of exumbrella arclength for two full cycles. The arclength is in percentage of the total relaxed exumbrella arclength. Error bars show the first variance. Peaks and valleys represent potential node locations.

Curvature was calculated for each point on each profile and then averaged over the 52 profiles. The average curvature for the right side of the bell is shown in Fig. 8 along with the first standard deviation. The peaks and valleys correspond to the highest deformation on average. The standard deviation indicates to what extent deformation occurred during swimming.

The average curvature curve shows that there are seven main peaks and valleys at 10, 24, 36, 66, 76, 84 and 96% of exumbrella arclength starting at the apex. These peaks and valleys correspond to potential node locations. The points corresponding to these peaks and valleys were found by identifying the points where Eq. 3 is closest to zero. Comparing the seven potential node locations with the location of the anatomical features we can see that three of the locations match well. The point at 36% matches with the beginning of the circular muscle start location with 4% difference, the point at 66% matches with the beginning of the radial muscles start location with a 2% difference and the point at 84% matches with the flap start location with a 4% difference.

Most points along the active part of the bell undergo a similar standard deviation. Variation increases significantly near the point at 82%. Comparing with the anatomical measurements, we can see that this point is close to the 88% point which marks the end of radial muscles and the beginning on the flap. The flap corresponds to a passive part of the bell beginning at the end of the swimming muscles up to the bell margin. The flap undergoes large deformation and movement during swimming but areas on the flap should not be considered node locations from a robotic perspective since this deformation can be achieved by a passive material [40]. The root of the flap which was found to be at 84% with the curvature analysis and 88% with the anatomical analysis is considered as a node location. The discrepancies between the anatomical and curvature node location may be due to several factors. Anatomy ratio variation with size is unknown for *C. capillata*. It is possible that the anatomical ratios measured varied between the two animals analyzed. Discrepancies in the footage processing can also have led to error in curvature analysis. Since a top view of the subumbrella was used to extract the anatomical information, the actual arclength of the subumbrella could not be used which led to error when calculating the arclength percentage location.

### Node Location Optimization

When modeling the kinematics of the *C. capillata*, it is desired to reduce the error between the natural profiles and the model. Depending on the application, the best number of segments used to represent the motion might differ. The error analysis technique was used to find which node location on the exumbrella profile minimized error for a given number of segments. This was done for 1 to 6 segments and the results are shown in Table 1.

**Table 1:** Node Locations.

	Node Location (%)							
Model	1	2	3	4	5	6	7	E
One-Seg	0	100						8.30
Two-Seg Opt	0	60	100					2.10
Three-Seg Opt	0	32	66	100				1.11
Four-Seg Opt	0	26	60	80	100			0.63
Five-Seg Opt	0	24	50	66	84	100		0.41
Six-Seg Opt	0	22	42	58	70	84	100	0.29
Four-Seg Anat	0	40	64	88	100			0.74
Four-Seg Curv Opt	0	36	66	84	100			0.75

Node locations found by the error analysis for discrete models representing the *C. capillata* exumbrella geometry. The associated error found by the error analysis between processed profiles and exumbrella model are listed. The four-segment anatomical model and four-segment curvature optimized model are also given with associated error. Node locations are given in exumbrella half arclength percentages starting from the bell apex.

**Figure 9:**
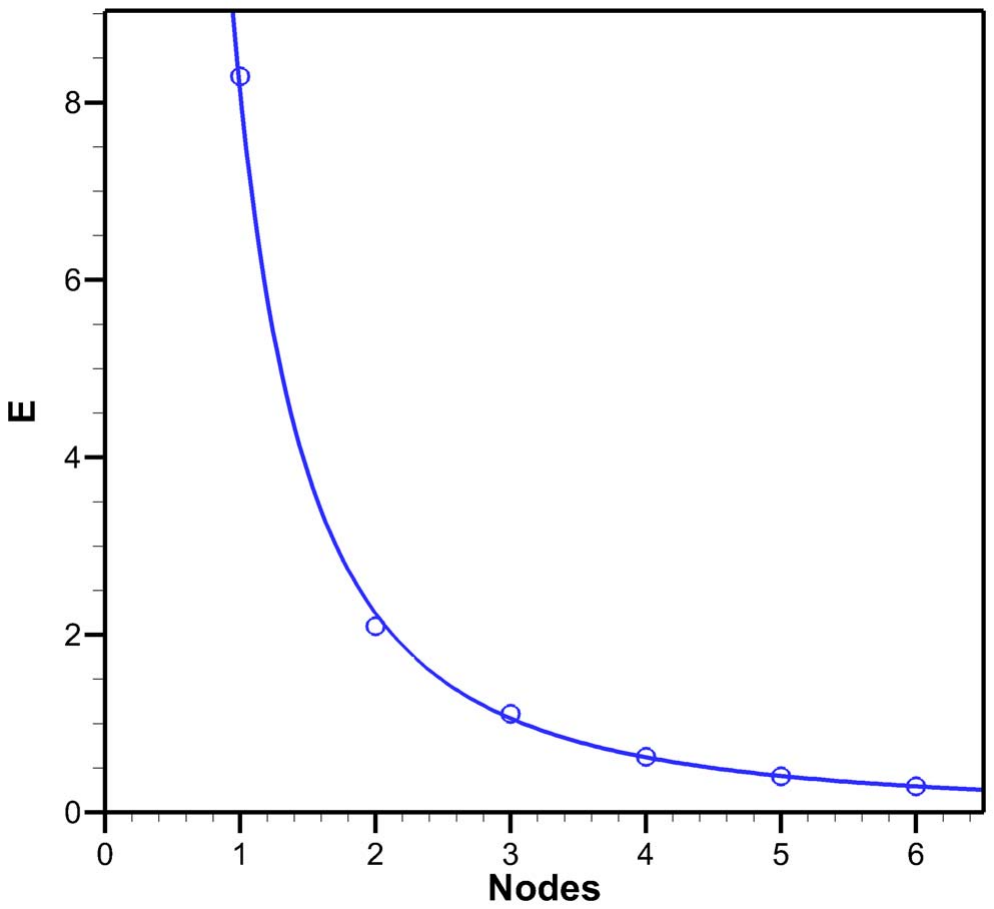
Error as a function of segments. Error (E) for different number of segments found by the error analysis method. The data has been fitted with power curves with equations 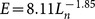 with a coefficient of determination of 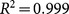 for the optimized data.

As expected, the error decreases with increasing number of segments. This is illustrated in Fig. 9. The relationship between error and increasing segment number has the following power function: 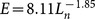 with 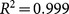 where 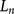 is the number of segments. Node location has a small effect on error as compared to the number of nodes. The four-segment optimized model along with the original *C. capillata* profiles are shown in Fig. 5. The seven possible node locations found by the curvature method were optimized to find which node combination results in the least amount of error for a four-segment model. The results are shown in Table 1 along with the node locations found from the anatomical analysis. Out of the three different four-segment models, the anatomical results had an error of 0.74, the curvature optimized method yielded the highest error with 0.75 and the fully optimized results had the lowest error with 0.63.

### Kinematic Model

Kinematic models of the four-segment optimized model and the four-segment anatomical model were developed using Fourier series. Segment length 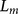 and angle between segments 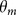, were fitted with Fourier series. The fit was done on the second swimming cycle. This cycle was clipped and shifted in time so that the contraction was followed by cruise and relaxation. It was then repeated nine times and fitted with a Fourier series. The Fourier coefficients for both kinematic models are shown in Tables S1-S4, see S1 Appendix. Using these coefficients, one can recreate the bell kinematics of a *C. capillata* approximated by four segments for any number of cycles using the following equations:
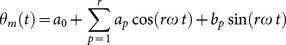
(24)

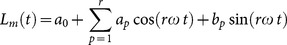
(25)where 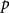 is the order of the Fourier transform used and 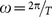 is the angular frequency where 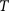 is the period of the swimming cycle.

### Discretized Bell Kinematics Analysis

**Figure 10:**
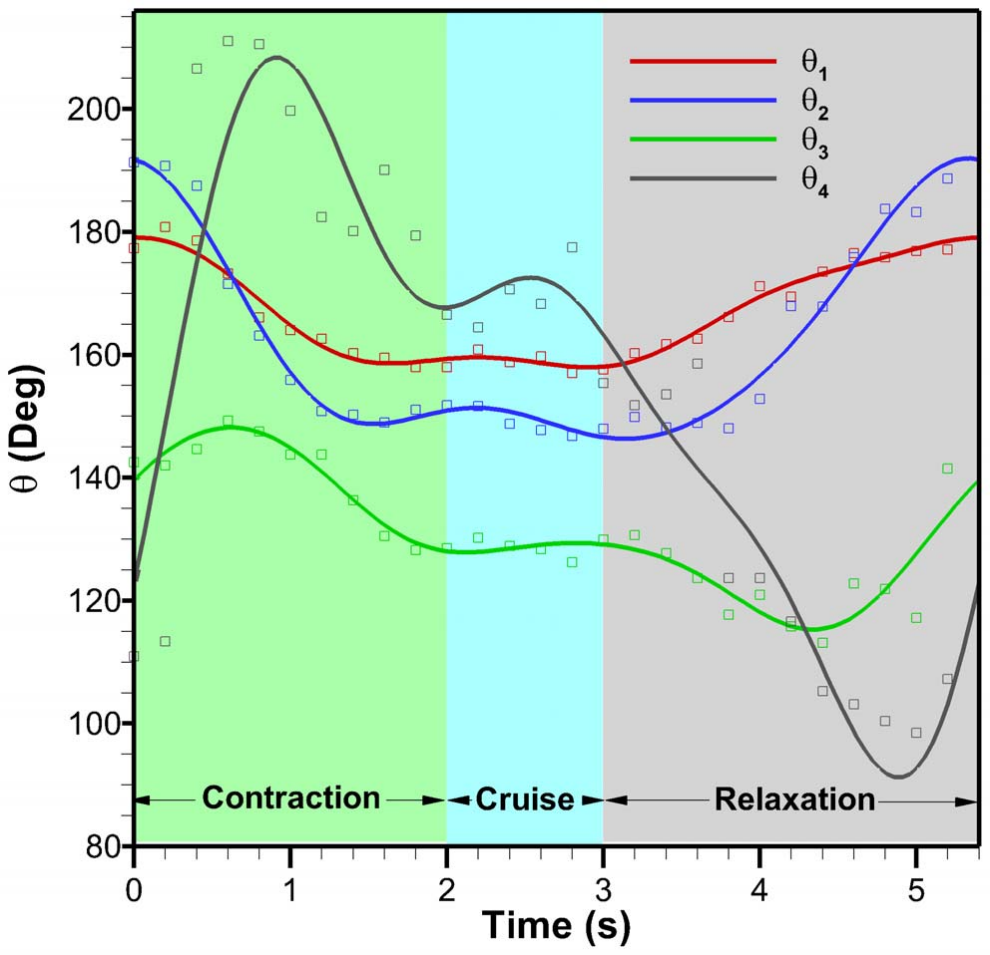
Segment angles as a function of time. Angles between adjacent segments of the four-segment anatomical model over a full cycle. Squares represent the four-segment anatomical model and lines represent the four-segment anatomical Fourier model.

A discrete representation of the *C. capillata* bell allows for an analysis of individual bell sections. The four-segment anatomical Fourier model is used to help gain an understanding of how the different anatomical regions behave during a swimming cycle. The angles formed between each segment are plotted as a function of time in Fig. 10 for one full cycle. The second cycle of the *C. capillata* was used for this analysis and is also the same cycle used for the Fourier model. The original data was clipped and shifted in time so that the contraction occurs first instead of relaxation. Fig. 10 also shows the corresponding four-segment anatomical model.

Contraction is taken as the period when the active sections of the bell are moving inwards. This occurs when the circular or radial muscles cause the bell to contract. Fig. 10 shows that the contraction lasts for 2 s which ends when the radial muscles stop moving the bell inward. Following contraction, the bell undergoes little movement for 1 s. This period is referred to as cruise time. It is unknown if the muscles are still actuating during cruise time or if the hydrodynamic forces are enough to keep the bell contracted. Cruise is followed by the relaxation phase where the elastic energy stored in the bell allows the bell to return to its fully relaxed position. Relaxation is first seen in Segments 1 and 2 and lasts for 2.4 s. Segment 3 sees further inward motion initially and then starts relaxing. The total cycle time for this 50 cm in diameter jellyfish is 5.4 s for an actuation frequency of 0.19 Hz. Counting the cruise time as the relaxation phase, the duty cycle is 38% which is similar to other rowing jellyfish which range from 32% to 42% [1], [15], [20], [22], [41]. With cruise time part of the contraction phase, the duty cycle is 58% which would be the duty cycle used if actuators are powered to maintain deformation.

In the four-segment anatomical Fourier model of Fig. 5, Segment 1 represents the central disk, Segment 2 represents the circular muscles, Segment 3 represents the radial muscles and Segment 4 represents the passive flap. Fig. 1B shows a schematic of segment locations and associated anatomical features which are summarized in Table 2 along with their functions during swimming. The contraction phase is initiated by the circular muscles at which point the radial muscles and flap are still expanding. The circular muscles undergo a total angle change of 42°. The radial muscles start contracting 0.6 s after the circular muscles and bend the bell by an additional 20°. Though the central disk is passive, it undergoes an angle change of about 20° due to contraction of the circular and radial muscles. Segment 4, which corresponds to the flap, undergoes significant lag during all phases of the swimming cycle. As angles 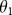 and 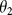 are decreasing during contraction, the flap angle 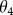 is increasing. About halfway during contraction, the flap angle starts decreasing. During relaxation, the flap angle decreases further until 5 s where it then starts increasing. This sudden increase is associated with the deceleration of segment 2 and allows the elastic energy in the flap along with its inertia to move the structure to its original position.

**Table 2:** Function of anatomical features with corresponding discrete model analog.

Anatomical Feature	Model	Motion	Contraction	Relaxation
Central Disk	Segment 1	Passive	20° ↓	20°↑
Circular Muscles	Segment 2	Active	42° ↓	46°↑
Radial Muscles	Segment 3	Active	8°↑, 20°↓ 0.6 s Lag	13°↓, 25°↑ 1.3 s Lag
Flap	Segment 4	Passive	85°↑, 41°↓ 0.9 s Lag	72°↓, 32°↑ 1.9 s Lag

The “Motion” column describes if the motion of a given feature is done passively or actively. “Contraction” and “Relaxation” columns describe the angle change during each phase and the associated lag period if any. Cruise was omitted from the table since negligible angle change occurs.

**Figure 11:**
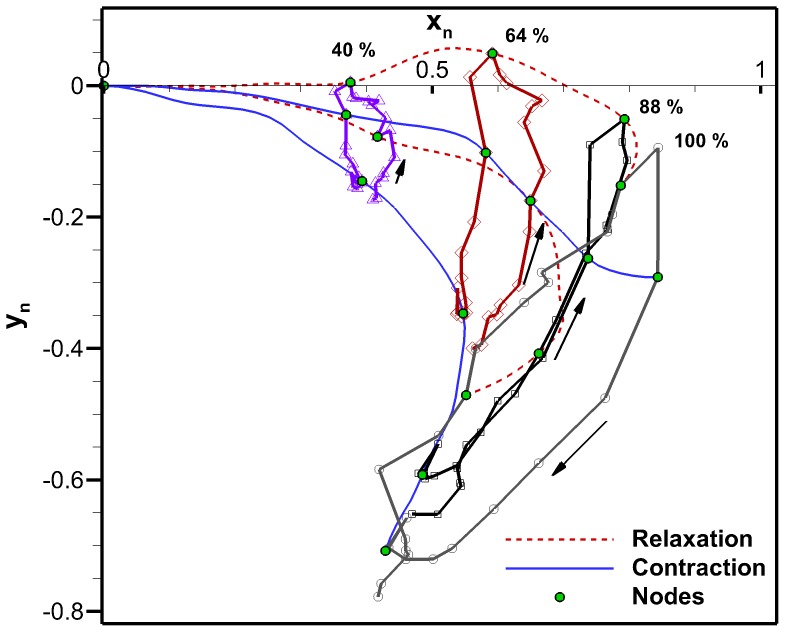
Four-segment anatomical model bell trajectory. Bell trajectory at node locations of the four-segment anatomical model during the second swimming cycle.

**Figure 12:**
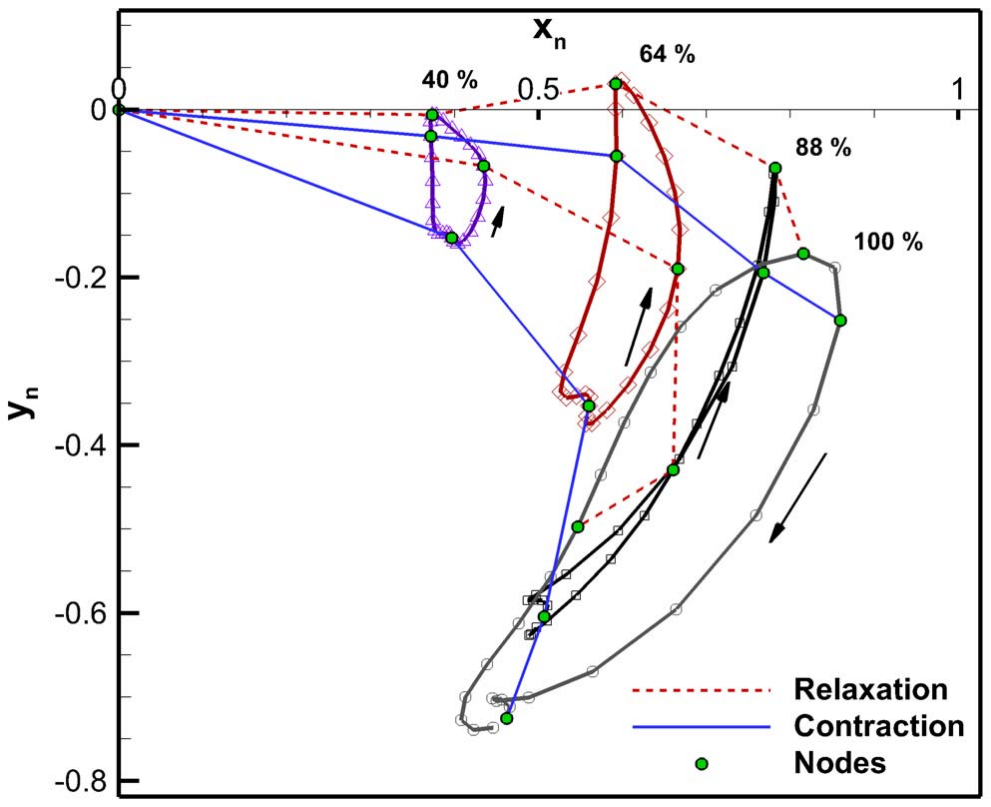
Four-segment anatomical Fourier model bell trajectory. Bell trajectory at node locations of the four-segment anatomical Fourier model during the second swimming cycle.

The bell trajectories at the anatomical node locations during a full swimming cycle are plotted in Fig. 11. Each node trajectory undergoes looping between contraction and relaxation. The bell margin consisting of node location 100% has the largest looping. The margin contracts with outer trajectory and relaxes with an inner trajectory. The 88% node undergoes almost no hysteresis during most of the cycle. Nodes 64% and 40% have hysteresis and their trajectory is inner during contraction and outer during relaxation. The flap therefore turns an inner trajectory during contraction into an outer trajectory and vice versa during relaxation. An outer trajectory during contraction increases the surface area used to produce thrust while an inner trajectory during relaxation reduces the surface area subjected to drag forces. These trajectory patterns are consistent with observations made on *Aurelia aurita* [23]. Fig. 12 is the equivalent plot for the four-segment Fourier model. The segment angles for this plot are the same as shown in Fig. 10. The Fourier model trajectories have the same behaviors as the original *C. capillata* kinematics.

### Subumbrella Volume Change

**Figure 13:**
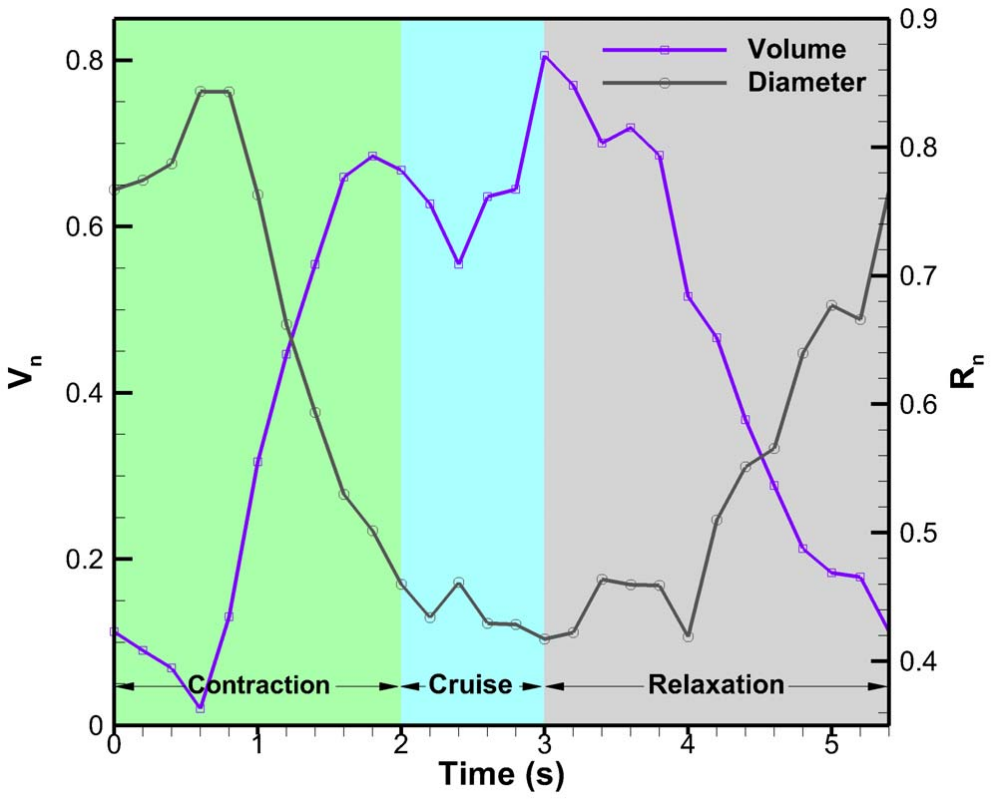
Subumbrella volume. Subumbrella volume and bell radius as a function of time for one full swimming cycle. The diameter is taken as the x-position of the bell margin.

The subumbrella volume and bell diameter as a function of time for one full cycle are plotted in Fig. 13. The subumbrella volume is approximated using the exumbrella profiles and the bell diameter is taken at the bell margin. The results in Fig. 13 show that the volume decreases briefly as the *C. capillata* begins to contract. This is due to the bell extending and flattening as contraction begins. When the radial muscles start contracting at 0.6 s, the subumbrella volume then starts increasing. The volume fluctuates slightly while cruising and starts decreasing during relaxation. This goes against the widely accepted jetting model used to quantify jellyfish propulsion [39]. Bell diameter as a function of time is inversely proportional to the volume of water enclosed by the subumbrella. The cycle shown in Fig. 13 is the same as in Fig. 10.

## Discussion and Conclusions

Many large or delicate marine animals are poorly studied and understood due to our lack in ability to acquire accurate data about them in their natural environment. The ability to use in situ recordings to obtain accurate kinematics provides insight into how these animals interact with their environment as well as potential for growth and reproduction. For jellyfish, swimming not only provides locomotion but also affects prey encounter [42]. However, very little is known about the influence of larger species. Therefore, understanding kinematics of swimming in these large specimens will provide insight into their influence on trophic ecology within marine ecosystems. It will also give insights on how jellyfish kinematics vary with size [11]. This affects hydrodynamics and therefore thrust and efficiency. The kinematics can also be used to model the fluid response of the animal's motion and the animal's structural response to the fluid interaction. Jellyfish hydrodynamics can be studied directly from live specimen but the analysis is limited since it is often not feasible to change parameters on a live animal. Having a kinematic model of an animal allows for the manipulation of many physical parameters that can guide the design of experiments as well as computational analysis [30], [31], [43], [44]. The in situ image correction techniques and the kinematic analysis methods developed in this work as led to the development of a *Cyanea capillata* biomimetic robot named Cyro [45]. Cyro measures 1.7 m in diameter, weighs 76 kg and is able to swim autonomously. A second robot named Cyro 2 is currently being developed to match the kinematics found in this work.

Only one plane was analyzed for the *C. capillata* kinematics since the information we were interested in is contained in that plane. This is a result of the animal's muscle structure and geometry. For more complex motions and geometries, the same techniques can be applied to additional view angles and combined to give kinematic information on different planes. This can help quantify motion that occurs in three dimensions such as the oscillation and undulation of a manta ray's pectoral fin [28] or the overall motion of a trout [46]. When multiple view angles are utilized, common reference points must be used to properly orient the kinematics. Some assumptions can be made based on the animal's known geometry. For example, if a front and side view of the animal are analyzed, and assuming criteria (1)–(3) are met, then only one reference point is needed to mark where the two planes intersect. An offset can be applied to the planes as needed. Ultimately, the kinematics of an animal can be reconstructed from image sequences the same way as geometries can be reconstructed using computer vision techniques such as photogrammetry [47].

The bell segment facing the camera was tracked and compared with the bell apex to see if there was rotation about the x- and y-axis. The results showed that rotation was in the same range as the error from image tracking and was therefore neglected. The three node detection methods presented in this study offer different benefits. The curvature method determines where the areas of highest deformation occur. With this method, there is no need to actually look at the animal's anatomy. Footage of animal kinematics satisfying the three selection criteria is the only information required. The error analysis method provides the best node and segment representation of the exumbrella for a given number of segments. This can be especially useful for modeling the bell in computational applications as a discrete representation. On the other hand, it does not take into account the mechanics of the bell which would be necessary in a robotic application. To reproduce the same kinematics and morphology of the *C. capillata*, the material behavior and mechanics must also be taken into account. For such application, the dimensions given by the anatomical analysis might have better results. This method shows where artificial actuators should be located if they were able to recreate the same deformation assuming they have the same form factor as the natural muscles. A downside of this method is the required analysis of the natural animal's anatomy which may not be feasible. The kinematic models provided can be used as a time dependent representation of the *C. capillata* bell motion. The four-segment optimized kinematic model best matches the exumbrella kinematic representation for four segments. The four-segment anatomical kinematic model provides the kinematics of the bell at locations where important anatomical features act.

The four-segment anatomical Fourier model revealed important features of the *C. capillata* bell kinematics. It was found that the bell does not deform uniformly. The radial muscles actuate with a lag period from the circular muscles and also lag behind the circular muscles during relaxation. The passive flap was found to have significant lag behind all the other sections. This leads to looping in the bell margin causing the bell to have an outer trajectory during contraction and inner trajectory during relaxation. The kinematics analysis also revealed that the *C. capillata* had a cruise time which lasts for 1/5^th^ of its swimming cycle where the bell geometry stays near constant. Rowers such as the *A. aurita* do not undergo cruise time. Their contraction is followed by an immediate relaxation [1], [15], [20], [22]. Jetters, conversely, usually undergo a cruise time after full relaxation. Cruise time after contraction has shown to increase the performance of a segmented bell Robojelly [6]. This leads to the hypothesis that cruise time improves the performance of segmented bell jellyfish. Cruise time may also be a function of size. The *C. capillata* models found in this study coupled with a hydrodynamic analysis will allow for the investigation of these hypotheses.

Rowing jellyfish propulsion has been modeled as a combination of jetting and paddling [12], [16], [17]. Jetting is a function of subumbrella volume change between the relaxed state and fully contracted state [39]. However, jetting depends on a decrease in subumbrella volume during contraction which is not the case for the *C. capillata* as was found in this analysis. According to the jetting model this should result in a negative thrust. A jetting model is therefore inappropriate for representing the propulsion mechanism of *C. capillata*. The starting and stopping vortex interaction between the contraction and relaxation phase described by Dabiri et al. [16] is likely to play a more prominent role in the thrust production of *C. capillata*.

An important aspect of *C. capillata* bell kinematics which requires further exploration is the kinematics of individual bell segments. Bell segments have a three-dimensional deformation which comes from the presence of a passive flap that surrounds the circumference of each bell segments. Also, the tentacles and oral arms being a prominent mass portion of the animal will influence the dynamics and therefore kinematics of the animal. The extent to which they contribute to the kinematics will require further investigation.

## Supporting Information

S1 Appendix **Fourier transform coefficients.**
(DOCX)
