# Supplementary material for: Cyanea capillata Bell Kinematics Analysis through Corrected In Situ Imaging and Modeling Using Strategic Discretization Techniques
Source: PLoS One. 2014 Dec 26;9(12):e115220. doi: 10.1371/journal.pone.0115220 (PMC4277286; doi:10.1371/journal.pone.0115220)
Supplement: S1 Appendix — Fourier transform coefficients. (DOCX) [file pone.0115220.s001.docx]

**Appendix**

The Fourier transform coefficients for the four-segment anatomical Fourier model and four-segment optimized Fourier model are shown in Tables S1-S2. The coefficients are for the angles formed between each segments and segment lengths for one full swimming cycle. These coefficients can be used along with Eqs. 8 and 9 to reconstruct the kinematics of a swimming *C. capillata*.

Table S1: Fourier transform coefficients for the angles of the four-segment anatomical kinematic model.

| **Segment** | **a_0_** | **a_1_** | **b_1_** | **a_2_** | **b_2_** | **a_3_** | **b_3_** | **a_4_** | **b_4_** |
| --- | --- | --- | --- | --- | --- | --- | --- | --- | --- |
| 1 | 1.72E+02 | -5.56E+00 | 2.73E+00 | 1.33E+00 | -6.17E-01 | -5.56E-01 | -8.78E-01 |  |  |
| 2 | 1.63E+02 | -1.82E+01 | 6.25E+00 | 4.86E+00 | -3.83E+00 | -1.91E+00 | -1.49E+00 |  |  |
| 3 | 1.38E+02 | -1.06E+01 | -2.39E+00 | 6.05E+00 | 6.03E-01 | -2.53E-01 | 2.52E+00 | 6.75E-01 | -1.05E+00 |
| 4 | 1.44E+02 | 7.01E+00 | -3.53E+01 | 2.77E+00 | 2.08E+01 | 1.69E+00 | -8.94E+00 |  |  |

Table S2: Fourier transform coefficients for the angles of the four-segment optimized kinematic model.

| **Segment** | **a_0_** | **a_1_** | **b_1_** | **a_2_** | **b_2_** | **a_3_** | **b_3_** | **a_4_** | **b_4_** |
| --- | --- | --- | --- | --- | --- | --- | --- | --- | --- |
| 1 | 1.72E+02 | -5.56E+00 | 2.73E+00 | 1.33E+00 | -6.17E-01 | -5.56E-01 | -8.78E-01 |  |  |
| 2 | 1.63E+02 | -1.82E+01 | 6.25E+00 | 4.86E+00 | -3.83E+00 | -1.91E+00 | -1.49E+00 |  |  |
| 3 | 1.38E+02 | -1.06E+01 | -2.39E+00 | 6.05E+00 | 6.03E-01 | -2.53E-01 | 2.52E+00 | 6.75E-01 | -1.05E+00 |
| 4 | 1.44E+02 | 7.01E+00 | -3.53E+01 | 2.77E+00 | 2.08E+01 | 1.69E+00 | -8.94E+00 |  |  |

Table S3: Fourier transform coefficients for the lengths of the four-segment anatomical kinematic model.

| **Segment** | **a_0_** | **a_1_** | **b_1_** | **a_2_** | **b_2_** |
| --- | --- | --- | --- | --- | --- |
| 1 | 0.4119 | 0.02857 | 0.01864 | -0.00941 | 0.001599 |
| 2 | 0.2472 | 0.02003 | 0.009608 | -0.00518 | 0.001316 |
| 3 | 0.2396 | 0.02259 | -0.00189 | -0.0022 | 0.006168 |
| 4 | 0.1231 | 0.01133 | 0.005077 | -0.004 | -0.00047 |

Table S4: Fourier transform coefficients for the lengths of the four-segment optimized kinematic model.

| **Segment** | **a_0_** | **a_1_** | **b_1_** | **a_2_** | **b_2_** |
| --- | --- | --- | --- | --- | --- |
| 1 | 0.27 | 0.0214 | 0.01106 | -0.00562 | 0.001065 |
| 2 | 0.3521 | 0.0281 | 0.013 | -0.00769 | 0.000486 |
| 3 | 0.2034 | 0.01421 | 0.005412 | -0.00345 | 8.30E-05 |
| 4 | 0.1995 | 0.02512 | 0.002363 | -0.00568 | 2.00E-03 |
